# Agreement between clinical measures to classify foot posture in asymptomatic adults

**DOI:** 10.1186/s12891-022-06023-z

**Published:** 2022-12-01

**Authors:** Torkamol Hunsawong, Phornchanok Motantasuta, Lugkana Mato, Wanida Donpunha

**Affiliations:** 1 Research Center in Back, Neck, Other Joint Pain and Human Performance (BNOJPH) Khon Kaen University, Khon Kaen, Thailand; 2 School of Physical Therapy, Faculty of Associated Medical Sciences, Khon Kaen University, Khon Kaen, Thailand

**Keywords:** Foot arch, Foot posture index-6, Foot type classification, Normalised navicular height truncated

## Abstract

**Background:**

Various clinical measures of static foot posture have been developed and used. However, consensus among clinical measures to classify foot posture remains to be established. Therefore, this study aimed to determine the level of agreement as a reliability component between two common clinical methods in asymptomatic adults: the normalised navicular height truncated (NNHt) and the Foot Posture Index-6 (FPI-6).

**Methods:**

The NNHt and FPI-6 were conducted on 102 asymptomatic adults. The measurement sequence was randomly arranged for each participant. Weighted Kappa (*K*_*w*_) was used to determine the agreement between the methods.

**Results:**

Both the NNHt and FPI-6 achieved similar foot posture distributions: approximately 40–50% of the participants had a normal foot, approximately 40% had a pronated foot and approximately 10–20% had a supinated foot. The agreement between the methods to classify foot posture was excellent (*K*_*w*_ = 0.84).

**Conclusions:**

The present study found excellent agreement between two commonly used clinical measures. This finding highlights the NNHt and FPI-6 consensus for foot posture classification in asymptomatic adults.

## Background

Human foot posture can be classified according to whether an individual has normal (neutral-arched), pronated (low-arched, flat or pes planus), or supinated (high-arched or pes cavus) feet [1]. Pronated or supinated feet are associated with altered lumbosacral alignment and function in the lower extremity, according to the joint coupling and closed-kinematic chain model [2–4]. Furthermore, pronated or supinated feet have been recognised as a predisposing factor for exercise-related lower limb injuries and chronic nonspecific low back pain [2–4]. Therefore, a screening assessment of foot posture should be conducted in asymptomatic individuals. This will lead to knowledge of selecting appropriate shoes for each individual, as well as exercise programs or foot orthoses for pronated or supinated feet to prevent sports-related lower limb injuries and low back pain. To manage foot-related lower extremity disorders and low back pain, it is important to have a valid, feasible and reliable quantitative clinical measure to differentiate and diagnose normal and abnormal foot postures.

Based on the anatomical alignment and/or anthropometrics of the foot, clinical non-radiographic measurement methods and parameters have been developed and commonly used to classify static foot posture and establish foot-related musculoskeletal disorders in clinics and research studies [1, 4, 5]. Reliability and validity studies have approved of such methods and parameters [6–8]. Of them, the navicular height/truncated foot length, also known as the normalised navicular height truncated (NNHt) has demonstrated moderate to strong correlations with radiographic evaluations [9], with good to excellent intra- and inter-reliability [8]. Among common clinical measurements, NNHt emerged as the most useful tool for determining the skeletal alignment of the medial longitudinal arch [10]. In addition, the NNHt is a simple, feasible and quantitative method. The NNHt, on the other hand, is primarily a uni-planar foot classification method that focuses on the sagittal plane of the foot and may not represent a complete tri-planar foot posture.

In the clinical setting, the most commonly used measure to classify foot posture is tri-planar visual observation, which is a qualitative examination that depends on the examiner’s experience [1, 7]. To establish a more reliable and reproducible measure, the Foot Posture Index-6 (FPI-6) was developed as a semi-quantitative assessment tool that combined measurements of the frontal, sagittal and transverse planes of the foot [11]. The FPI-6 has demonstrated a strong correlation with electromagnetic tracking methods that can predict subtalar position, a good validity with a static lower limb kinematic model, excellent intra-rater reliability [11–14] and moderate inter-rater reliability [13]. Accordingly, the moderate inter-rater reliability suggests that the FPI-6 should be used with extreme caution [13]. The FPI-6 user guide and manual recommend that the novice assessor rate at least 30 individuals with as many different foot postures as possible after receiving intensive training before using the FPI-6 in clinics and research studies [12].

The NNHt and the FPI-6 serve as common clinical measurements to classify foot posture in terms of validity, feasibility, and reliability. However, each measure may reflect different aspects of arch structure and thereby classify foot posture into different types. In essence, each foot posture assessment method differs in terms of cut-off points and scales used to classify foot posture, which can lead to inconsistencies and make it difficult to compare, generalise, and pool these results across research studies. Therefore, agreement analysis is critical for developing a standardised approach to the topic.

Literature search showed that only one study had conducted an agreement analysis of the clinical methods for static foot posture classification [14]. This study demonstrated moderate level of agreement amongst the methods most commonly used in their institute to classify foot posture in 30 asymptomatic adults: the rearfoot angle, medial longitudinal arch angle, navicular drops and FPI-6 [14]. The authors noted that fair intra-rater reliability for methods such as the navicular drops could influence on level of agreement [14]. A previous study reported a significant correlation between the raw scores of the NNHt and the original FPI-8 for foot classification in older adults [10]. The study determining the consensus between the NNHt and the modified current version — the FPI-6 to classify foot posture, remains to be established. Consequently, an agreement analysis of the recommended valid and reliable measurements, the NNHt and FPI-6 is still needed. Therefore, the present study aimed to determine the level of agreement between the NNHt and the FPI-6 to classify foot posture in asymptomatic adults.

## Methods

### Participants

This cross-sectional descriptive study targeted individuals, male or female, aged 18–45 years with a body mass index (BMI) ranging from 18.5–24.9 kg per square metre (kg/m^2^) who were able to perform and follow research instructions. Participants were excluded if they were pregnant and/or had lower-extremity pain or injury within the six months prior to data collection, current lower extremity pain, a history of surgery and/or fracture of the spine or lower extremity, diagnosed neurological deficits such as stroke and spinal cord disorder, spinal scoliosis, rheumatoid, gout and/or systemic lupus erythematosus [9, 14, 15]. The study protocol was approved by the local centre for ethics in human research (Registration number: HE602301). Individuals who live in local province were invited to participate in this study using an advertisement (i.e., a poster) and face-to-face meetings. Prior to data collection, all potential participants gave their written informed consent.

### Sample size calculation

The optimal number of participants was calculated based on an agreement analysis of two different measures with Cohen’s kappa and aimed to detect a significant substantial agreement (kappa = 0.80) between them. To obtain the optimal sample size, the present study consulted the table for sample size estimation for kappa analysis [16]. The present study defined the kappa to detect as approximately 0.80, with the null hypothesis value of kappa equal to 0.40 (two-tailed) and the power of the test set to 80%. Hence, the optimal sample was 102 individuals.

### Clinical measures of foot type classification

The level of agreement was determined using the right foot of all participants [14, 17]. The measurement procedures were conducted as follows.

**Fig. 1:**
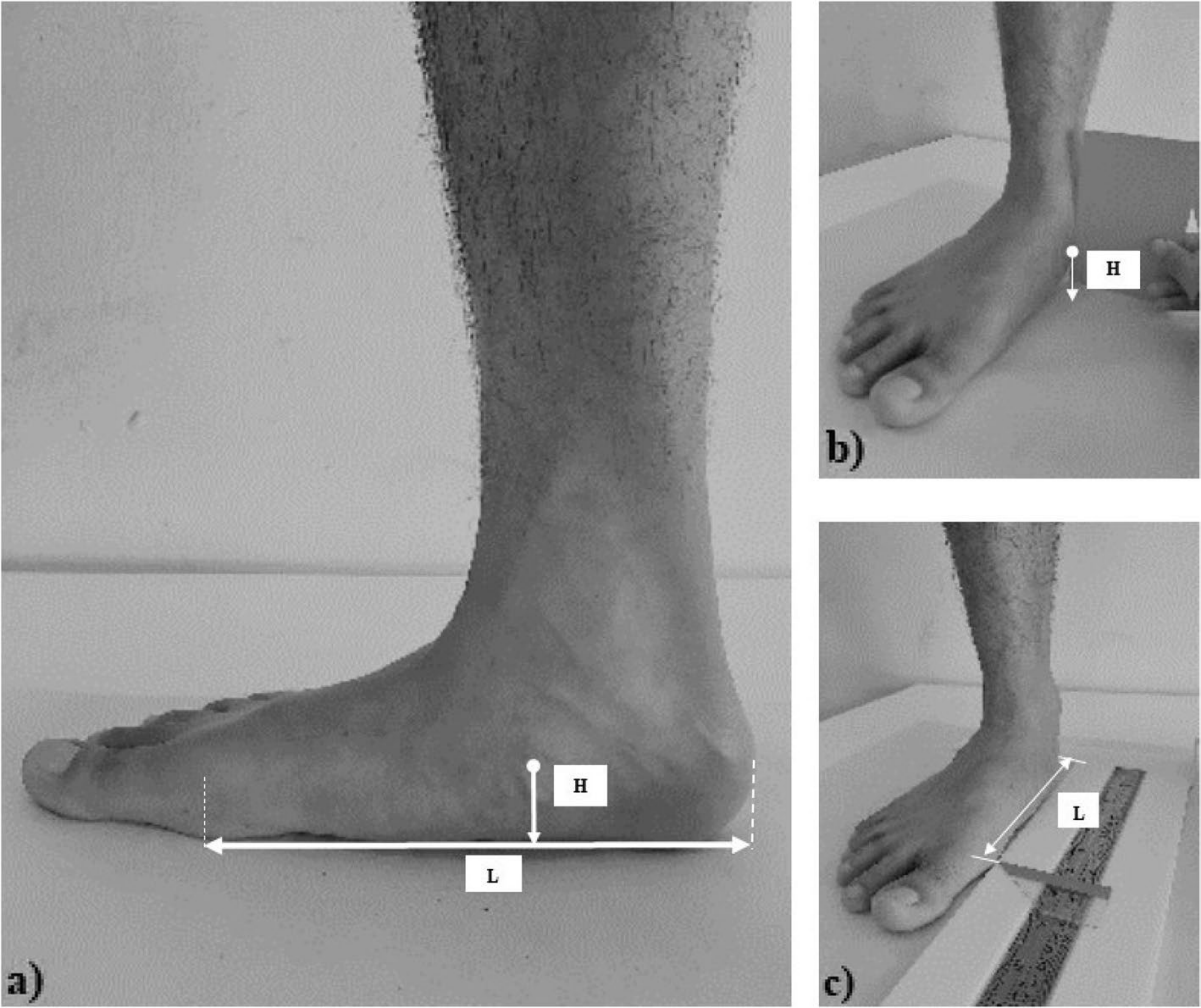
Anatomical landmarks for the normalised navicular height truncated (NNHt) calculation. **a**) NNHt = H/L, **b**) the perpendicular distance between the most prominent part of the navicular tuberosity and the supporting floor (H), **c**) the distance between the first metatarsophalangeal joint and the most posterior end of heel (L)

The NNHt (Fig. 1) was conducted as the uni-planar measure to classify normal, pronated and supinated feet [9, 18]. The NNHt referred to the ratio of the navicular height (mm) to the truncated length of the foot (mm). While barefoot, participants were asked to stand still with their arms by their sides in the double-stance relaxed position on top of a 90x80x25-cm stool. The perpendicular distance between the most prominent part of the navicular tuberosity and the supporting floor (H) was measured using a card size of 7 × 12 cm^2^, and a dial calliper (precision = 0.05 mm; Oxford Precision, Oxford, GB) was used to measure the H. Then the truncated length of the foot (the perpendicular distance between the first metatarsophalangeal joint and the most posterior end of the heel) was measure using a steel ruler (L). Three repeat measures were conducted, and the average value was used to classify foot posture. Participants with NNHt scores < 0.17 had a highly pronated foot, whereas those with scores from 0.17–0.21 had a pronated foot, 0.22–0.31 a normal foot and 0.32–0.35 a supinated foot. Scores > 0.35 represented a highly supinated foot [9].

**Fig. 2:**
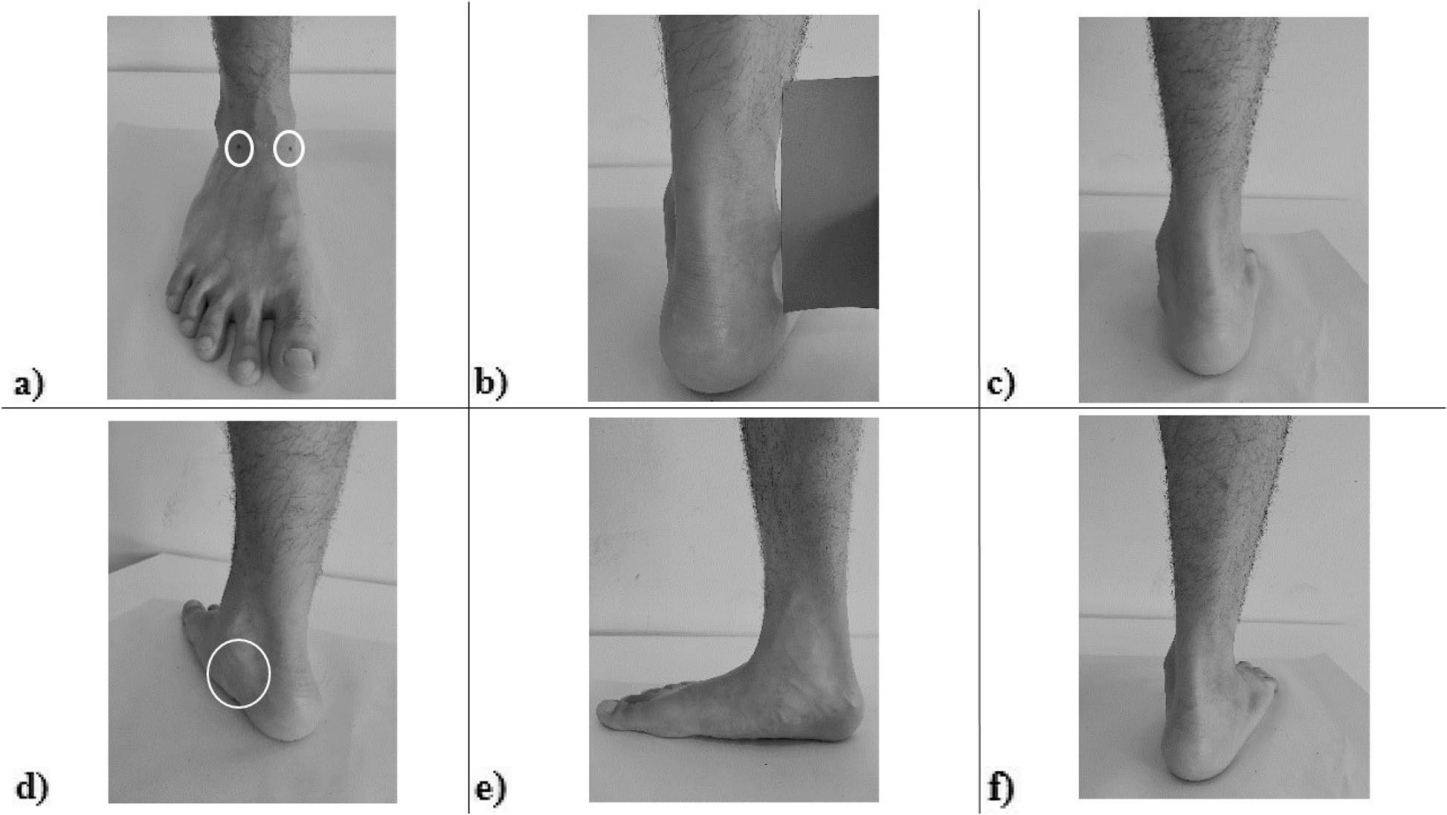
The six-criteria of Foot Posture Index-6. **a**) talar head palpation (white circles), **b**) lateral malleoli curvature, **c**) calcaneal inversion/eversion, **d**) talonavicular bulging (white circle), **e**) height and congruence of medial longitudinal arch, and **f**) forefoot on rearfoot abduction/adduction

The FPI-6 was conducted as the tri-planar measure that combined the semi-quantitative examination of foot posture from the forefoot, midfoot and rearfoot segments, and it can identify normal, pronated and supinated feet [11]. While barefoot, participants were asked to stand still for two minutes with their arms by their sides in the double-stance relaxed position. The rater observed and palpated the participant’s foot and scored each criterion of the FPI-6. The FPI-6 consists of six criteria: a) talar head palpation, b) lateral malleoli curvature, c) calcaneal inversion/eversion, d) talonavicular bulging, e) the height and congruence of the medial longitudinal arch, and f) the forefoot on rearfoot abduction/adduction (Fig. 2). Each criterion was rated on a 5-point score ranging from − 2 to + 2. A negative score represented a supinated foot, a zero score represented a normal foot, and a positive score represented a pronated foot [12]. The total scores ranged from − 12 to + 12 and were used to classify foot posture. Participants with total scores ≥10 had a highly pronated foot, whereas those with scores from 6 to 9 had a pronated foot, 0 to 5 a normal foot and − 1 to − 4 a supinated foot. Those with scores ≤ − 5 had a highly supinated foot [19].

### Procedure

A rater (a physiotherapist) with one year of extensive training in management for musculoskeletal disorders, including the use of the NNHt and FPI-6 measures [12, 20], conducted both methods to all participants. Prior to data collection, the inter- and intra-rater reliability of the NNHt and FPI-6 were determined in 30 asymptomatic adults (these participants were not recruited in the main part of the present study). For inter-rater reliability, two investigators participated in this step: the rater and an experienced physiotherapist who expertise in foot/ankle management with intensively conducted the NNHt and FPI-6 to classify foot posture. For the intra-rater reliability, the rater conducted both methods on the same day, with a 10-minute break between the methods. The results demonstrated excellent inter- and intra-rater reliability (ICCs: 0.98–0.99) [21].

For data collection, the eligible participants were asked to provide their personal demographic data by filling out a form (e.g. age, gender, weight and height). The rater conducted the static measurements for foot classification, with a 10-minute rest between the methods [14]. The measurement sequence was simply randomised for each participant. After completing all measures, the NNHt and FPI-6 scores were calculated to classify the foot type for each method.

### Statistical analysis

All data analyses were performed using Microsoft Excel 2016 (MicrosoftCorp, Washington, USA), SPSS version 26 for Windows (IBM SPSS Statistics, New York, USA) and Stata version 10 for Windows (StataCrop, Texas, USA). The baseline characteristics (e.g., age, weight and height) were represented as follows: mean ± standard deviation (SD). The nominal data (e.g., gender) were represented as numbers, percentages or proportions when appropriate. Prior to the statistical analyses, the normal distribution was assessed using the Kolmogorov-Smirnov test. The present data were normally distributed. The Weighted Kappa (*K*_*w*_) was used to determine the agreement between the methods. The weights were calculated and expressed in the equation below [14], with foot classifications being coded as highly pronated, 0.2; pronated, 0.4; normal, 0.6; supinated, 0.8; highly supinated foot, 1.0.

$$\textrm{Weight}={\left({\textrm{i}}_{\textrm{n}}-{\textrm{j}}_{\textrm{n}}\right)}^2$$

Where; i = row.

j = column in test-retest matrix.

Weighted Kappa *(K*_*w*_) was interpreted as ≤0.4 is fair, 0.41–0.6 is moderate, 0.61–0.8 is substantial, and > 0.8 is excellent agreement [22].

## Results

The agreement analysis was performed on 102 asymptomatic adults (37 males and 65 females). The mean age of the participants was 28 ± 7 years, weight was 59 ± 9 kg, height was 164 ± 9 cm, and body mass index was 22 ± 2 kg/m^2^.

The mean score of the NNHt was 0.23 ± 0.05 (ranged from 0.10 to 0.36), whereas the mean score of the FPI-6 was 4 ± 4 (ranged from − 7 to + 11). The results show that the NNHt and FPI-6 divided foot postures into similar categories. In terms of frequency, the majority of participants had a normal foot, followed by a pronated foot, a highly pronated foot, a supinated foot, and a highly supinated foot, respectively (Table 1). The present study demonstrated an excellent level of agreement (*K*_*w*_ = 0.84) between the NNHt and the FPI-6 by classifying foot posture in 102 asymptomatic adults (Table 1).

**Table 1:** Number of participants in each foot type and the Weighted Kappa analysis (*K*_*w*_)

Variables	Highly pronated foot	Pronated foot	Normal foot	Supinated foot	Highly supinated foot
**NNHt; n (%)**	10 (10%)	32 (31%)	54 (53%)	4 (4%)	2 (2%)
**FPI-6; n (%)**	13 (13%)	33 (32%)	39 (38%)	12 (12%)	5 (5%)
***K***_***w***_	0.84 (95%CI = 0.83–0.88; % Observed agreement = 99%)				

*Abbreviations*: *NNHt* the normalised navicular height truncated, *FPI-6* the foot posture index-6, *K*_*w*_ the Weighted Kappa statistic, *95%CI* 95% confidence interval: Total number of participants = 102

## Discussion

The present study reported the agreement between the uni-planar results from the NNHt and those tri-planar results from the FPI-6. The *K*_*w*_ demonstrated excellent agreement between the NNHt and the FPI-6. Regarding the plane of measurement in each item of the FPI-6, the height and congruence of the medial longitudinal arch address the sagittal plane; the lateral malleoli curvature and calcaneal inversion/eversion address the frontal plane; and talar head palpation, lateral malleoli curvature, talonavicular bulging and the abduction/adduction of the forefoot on the rearfoot address the transverse plane [11]. The three-dimensional nature of the foot structure may be represented by the uni-planar measurement obtained with the NNHt. Consequently, this may explain the excellent agreement reported in the present study. This result demonstrates a consensus between the NNHt and FPI-6 for foot posture classification in asymptomatic adults.

At present, only one previous study has conducted an agreement analysis of static foot posture methods, and the results are inconsistent with those of the present study [14]. This previous study investigated the level of agreement between the foot type classification methods commonly used in their institute using 30 asymptomatic adults [14]. Their result demonstrated a moderate agreement (Fleiss Kappa: *K*_*f*_ = 0.58) amongst the methods. In addition, this study reported moderate agreement between the test-retest results for the rearfoot angle (*K*_*w*_ = 0.60) and fair agreement and reliability between the test-retest results for the navicular drops (*K*_*w*_ = 0.40, ICC: 0.40). The authors suggested that the navicular drops may be an unreliable measurement method for classifying foot posture and could thus affect agreement analyses. The authors also noted that the agreement analysis may have been impacted by the small sample size and homogenous participants’ characteristics, given that the authors only recruited staff and students in their institute. Compared to this previous study [14], our study reports a higher level of agreement across the foot posture measurement methods, which may be due to the excellent reliability of the NNHt and FPI-6 for foot type classification.

The excellent agreement between NNHt and FPI-6 has clinical implications. This result indicates that the NNHt and FPI-6 can classify foot posture into similar categories (i.e., pronated, normal, and supinated feet) and demonstrate the consensus between the two measures for foot posture classification in asymptomatic adults. Overall, the present study’s results may help experts to develop a standardised approach to static foot posture assessment to pool and translate research findings into clinical practice (caution should be used when pooling data on the supinated foot from these methods).

There are some limitations that should be considered. First, this study was performed on asymptomatic adults aged from 18 to 45 years of age, which may limit the generalisability of the findings. Hence, further agreement research is needed in other age groups, such as children and the elderly, as well as patients with foot and ankle pathologies. Furthermore, having more raters in multicentre study and looking at inter- and intra-rater reliability would strengthen the clinical implication of the current findings. Second, the present study was performed on individuals with normal BMIs (18.5–24.9 kg/m^2^). Therefore, further agreement analyses are required to assess static foot posture in individuals with a variety of BMI ranges. Third, as the primary purpose of the present study was aimed to determine the level of agreement between clinical measures to classify static foot posture. Some would argue that clinical assessment of foot posture may not precisely compare to weight bearing radiographic measurement. Hence, further study should include radiographic evaluations as a reference. Finally, the present study reported a low number of participants with a supinated foot, which is consistent with the previous study. Therefore, further agreement analyses that focus on the supinated foot are still needed. Also, the responsiveness of the NNHt and FPI-6 has yet to be established. This will strengthen the use of these methods for monitoring the effectiveness of treatment and determining the patient’s prognosis.

## Conclusions

The present study investigated the level of agreement between the NNHt and the FPI-6 to classify foot posture in asymptomatic adults. The present findings demonstrated excellent agreement between the two methods. Based on the results, the NNHt and FPI-6 can classify foot posture into similar categories (pronated, normal and supinated feet). This finding highlights the consensus between the NNHt and FPI-6 to classify foot posture in asymptomatic adults.

## Data Availability

The datasets used and/or analyzed during the current study are available from the corresponding author on reasonable request.

## Abbreviations

BMI: Body mass index
FPI-6: Foot Posture Index-6
ICC: Intraclass correlation coefficient
*Kf*: Fleiss Kappa
kg/m^2^: kilograms per square metre
*K*_*w*_: Weighted Kappa
NNHt: Normalised Navicular Height truncated
SD: Standard Deviation
